# Influence of betel nut chewing on oral microbiome in Papua New Guinea

**DOI:** 10.1093/emph/eoae030

**Published:** 2024-11-09

**Authors:** Nicolas Brucato, Valentine Lisant, Christopher Kinipi, Alfred Kik, Guillaume Besnard, Matthew Leavesley, François-Xavier Ricaut

**Affiliations:** Centre de Recherche sur la Biodiversité et l’Environnement (CRBE), Université de Toulouse, CNRS, IRD, Toulouse INP, Université Toulouse 3 - Paul Sabatier (UT3), Toulouse, France; Centre de Recherche sur la Biodiversité et l’Environnement (CRBE), Université de Toulouse, CNRS, IRD, Toulouse INP, Université Toulouse 3 - Paul Sabatier (UT3), Toulouse, France; Strand of Anthropology, Sociology and Archaeology, School of Humanities and Social Sciences, University of Papua New Guinea, PO Box 320, University 134, National Capital District, Papua New Guinea; New Guinea Binatang Research Centre, PO Box 604, 511 Madang, Papua New Guinea; Biology Centre, Czech Academy of Sciences, 37011, Ceske Budejovice, Czech Republic; Centre de Recherche sur la Biodiversité et l’Environnement (CRBE), Université de Toulouse, CNRS, IRD, Toulouse INP, Université Toulouse 3 - Paul Sabatier (UT3), Toulouse, France; Strand of Anthropology, Sociology and Archaeology, School of Humanities and Social Sciences, University of Papua New Guinea, PO Box 320, University 134, National Capital District, Papua New Guinea; College of Arts, Society and Education, James Cook University, P.O. Box 6811, Cairns, Queensland, 4870, Australia; ARC Centre of Excellence for Australian Biodiversity and Heritage, University of Wollongong, Wollongong, New South Wales, 2522, Australia; Centre de Recherche sur la Biodiversité et l’Environnement (CRBE), Université de Toulouse, CNRS, IRD, Toulouse INP, Université Toulouse 3 - Paul Sabatier (UT3), Toulouse, France

**Keywords:** microbiome, *Areca catechu*, oceania, periodontal diseases, metagenomics

## Abstract

**Background and objectives:**

For thousands of years, betel nut has been used as a psychoactive agent in Asian and Oceanian populations. Betel nut chewing was associated with the alteration of human oral microbiome and with diseases such as oral cancer and periodontitis, but only in populations of Asian cultural background. We studied the influence of betel nut chewing on the oral microbiome in Papua New Guinea, where half of the population uses betel nut and the prevalence of these diseases is one of the highest in the world.

**Methodology:**

We characterized the oral microbiomes of 100 Papua New Guineans. We defined two cohorts of betel chewers (*n* = 50) and non-chewers (*n* = 50) based on a genetic approach to identify the presence of betel nut in saliva. We statistically compared the alpha and beta microbial diversities between the two cohorts. We performed linear discriminant analyses to identify bacterial species more prevalent in each cohort.

**Results:**

We found that oral microbial diversity is significantly different between betel chewers and non-chewers. The dysbiosis observed in betel chewers, led to an increase of pathogenic bacterial species including *Porphyromonas gingivalis*, *Treponema denticola,* and *Tannerella forsythia*, known to be in the aetiology of periodontal diseases.

**Conclusions and implications:**

Our study strongly supports the alteration of human oral microbiome by betel nut use, potentially leading to periodontal diseases. It also shows the need to consider local specificities (e.g. different habits, betel nut types, and oral microbial diversities) to better characterize the impact of betel nut chewing on health.

## INTRODUCTION

Betel nut is the fourth most common psychoactive agent worldwide after caffeine, alcohol, and nicotine [1]. This fruit of areca palm trees (*Areca* spp., Arecaceae) contains several alkaloids that can trigger euphoria, relaxation, and improved concentration [2]. Often chewed in combination with tobacco (*Nicotiana tabacum*) and the inflorescence of betel piper vine (*Piper betle*), betel nut is used by over 600 million people, especially in South Asia and Oceania where it has a strong significance in the expression of cultural and social identities [3]. Its most ancient use is archaeologically documented in Thailand around 8000 years ago [4]. Betel nut has long been used in traditional medicine for its potential anthelmintic effect and antibacterial activity [5]. Such a long-term use of betel nut could have influenced the evolutionary history of human populations, ultimately leading to specific biological diversities and adaptations [6, 7]. However, several studies have shown its deleterious impact on human health [8]. Betel nut chewing has negative physiological effects across multiple organ systems, including the nervous, cardiovascular, gastrointestinal, respiratory, endocrine, and reproductive systems [1]. It has been associated with several pathologies such as anaemia, oral cancer, and periodontal diseases [9–11].

In Papua New Guinea, betel nut chewing concerns almost half of the population, equally prevalent among men and women [12]. Locally known as ‘buai’ in *Tok Pisin*, betel nut (mainly *Areca catechu*) is usually chewed in combination with a vine fruit (mainly *Piper betle*, known as ‘daka’) and lime (known as ‘kambang’), usually without tobacco [13]. While the origins of the betel nut cultivation in Papua New Guinea remain unclear, there are archaeological and genetic evidence that *Areca catechu* has been spread in Papua New Guinea over the last three millennia, probably contemporaneously with the Austronesian dispersal [14–16]. Yet, wild local substitutes, such as *Areca macrocalyx* and *Piper gibbilimbum*, may have been used before [14]. Widely used in the coastal regions during the 1960s, areca palm trees are now frequently cultivated in the highlands as well [12]. It is currently grown in rural locations and traded across the country, representing a major source of income for communities. Apart from being perceived as ‘green gold for grassroots’ [13], betel nut is also regarded as a symbol of peace, traditionally used in peace ceremonies, bride price exchanges, and rituals to ward off evil spirits. However, betel nut chewing in Papua New Guinea has also been linked to several deleterious impacts on health, such as anaemia [17], reduced childbirth weight [18], and the spread of tuberculosis [19]. Papua New Guinean population has currently one of the highest prevalences of oral cancer and periodontal diseases in the world [8, 20].

A large part of the deleterious influence of betel nut chewing on human health would be due to its impact on oral microbiome homeostasis [11]. The oral microbiota is a major factor in human health [21]. It is composed of more than 700 bacterial species mainly belonging to six phyla: *Actinobacteria, Bacteroidetes, Firmicutes, Fusobacteria, Proteobacteria*, and *Spirochaetes* [22]. They form complex and distinct communities on teeth, tongue, hard pallets, and epithelial tissues, with the saliva representing a mixture [21]. Although they are very stable within an individual over time, external factors such as diet and other habits can alter their composition [7]. Several studies have found that betel nut chewing is a major cause of dysbiosis, potentially associated with oral cancer and periodontal diseases [23–26]. The oral microbiome diversity in Papua New Guinea presents specific features [27] that could be influenced by betel nut chewing, partly explaining the observed epidemiology [8, 20].

Here, we analysed oral microbiome data for 100 individuals from Papua New Guinea, including 50 betel chewers, in order (i) to characterize the oral microbial diversity in Papua New Guinean non-chewers; (ii) to determine the influence of betel nut chewing; and (iii) to identify potential pathogenic microbes associated with betel nut chewing.

## METHODOLOGY

### Ethics

This study was approved by the Medical Research Advisory Committee of Papua New Guinea (National Department of Health) under research ethics clearance MRAC 16.21 and by the French Ethics Committees (Committees of Protection of Persons CPP 25/21_3, n_SI: 21.01.21.42754). Permission to conduct research in Papua New Guinea was granted by the National Research Institute of Papua New Guinea (permit 99902292358), with full support from the School of Humanities and Social Sciences, University of Papua New Guinea. All samples were collected from healthy, unrelated adult donors who provided written informed consent.

### Dataset

We obtained 262 Papua New Guinean genomic data generated from saliva samples from publicly available datasets available on the European Genome-Phenome Archive (https://ega-archive.org/) [28, 29]: EGAD00001007783, EGAD00001010142, EGAD50000000050, and EGAD00001010143. All participants in these studies were healthy adults. The samples were collected using the Oragene OG-500 kit in four locations: Port Moresby, National Capital District; Daru Island, Western Province; Mount Wilhelm, Simbu Province, and Karawari River area, East Sepik Province. All genetic data were generated following the same shotgun sequencing protocol, at a depth of reading between 10× and 30× (Illumina HiSeq X5) (see for more details [28, 29]). We discarded all fastq reads mapping on the human reference genome GRCh38 (GCA 000001405.15) using GATK (reads mapping quality ≥ 20) [30].

Microbiome data were obtained from the fastq reads unmapped to the human genome using Kraken 2 [31] and the Standard plus protozoa and fungi database from GenBank (including archaea, bacteria, viruses, plasmids, humans, protozoa, and fungi; updated on 7 June 2022), as in Pedro *et al*. [27]. We defined a threshold of 10 reads as the minimum number of reads required for classification at the specified rank in each sample. The relative abundance of each taxa for each individual was calculated, after filtering a low number of reads, at the level of phyla, genera, and species separately. Three thousand one hundred fifty-seven known bacterial taxa were identified (Supplementary Table S1). We noted that only 1.4% of the reads were assigned to *Homo sapiens*, indicating a relatively efficient removal of the human genetic information. Bacterial species were classified as established pathogens according to Bartlett *et al*. [32], considering that this classification did not reflect necessarily their pathogenicity in the oral cavity. The relative abundances of each taxon were regressed for age and sex using a linear model, and residuals were used for all further analyses.

Since the genomic dataset was compiled from diverse studies, no information on betel nut use was available (i.e. no questionnaire on betel nut use was conducted). We used a genetic approach to determine the presence or absence of plants used in betel chewing by each individual [33]. We built a dataset of organellar DNA (chloroplastic and mitochondrial genomes) for all plant species endemic and imported to Papua New Guinea listed in [12, 34] and present in GenBank (*n*_species_ = 37 123; Supplementary Table S2). We constrained our dataset on organellar DNA since they are more abundant in a sample than nuclear DNA and to limit the bias of different genome lengths. For each individual shotgun sequencing data, we retrieved the fastq reads unmapped to the human genome and the microbiome using GATK [30] and Kraken 2 [31]. This set of reads was mapped to our plant organellar DNA dataset using Kraken 2. Considering that betel nut chewing in Papua New Guinea can also use local plants (e.g. *Areca macrocalyx* and *Piper gibbilimbum*) which are not characterized genetically, we considered mapping at the genus level (e.g. *Areca* and *Piper*). Our approach defined individuals as ‘betel chewers’ if at least 10 fastq reads mapped to *Areca* and *Piper*, or as ‘non-chewers’ if no read can be mapped to *Areca* or to *Piper*. We also reported the presence of tobacco (*Nicotiana tabacum*) and cannabis (*Cannabis sativa*), which were identified at the genus level for consistency.

### Statistical analyses

The alpha diversity within each sample, based on the relative abundance of all genera, was estimated by the Shannon and Inverse Simpson indices using the R package vegan v.2-6-4 [35]. *T*-tests were performed to evaluate the difference in alpha diversities between betel chewers and non-chewers. All statistical tests are corrected for multiple testing using the Bonferroni correction (*P*_*c*_).

The beta diversity between samples, based on the relative abundance of all genera, was estimated by the Bray–Curtis index using the R package vegan v.2-6-4. Principal coordinates analyses (PCoA) and non-metric multidimensional scaling analyses (NMDS) were computed to plot the beta diversities. Permutational Analysis of Variance (PERMANOVA) was performed to estimate the difference of beta diversities between the two groups (10 000 permutations).

We identified microbial genera significantly more present in one of the two groups using Linear discriminant analysis of Effect Size (LEfSE) [36]. For each genus giving a significant result in the Linear Discriminant Analysis (LDA), *t*-tests were performed at the species level between the two groups. A cladogram was computed, based on the phylogeny at the Domain, Phylum, Class, Order, Family, and Genus levels from the expanded Human Oral Microbiome Database (eHOMD; https://www.homd.org/ftp//phylogenetic_trees/genome).

## RESULTS

### Genetic identification of betel nut chewing

Among the 262 shotgun sequencing data available, we detected at least one of the plant genera characterizing betel nut chewing (*Areca* and *Piper*) for 125 individuals. It results in a ratio of 48%, close to the reported use of betel nut in the general Papua New Guinean population (around 50%) [12]. Cannabis (*Cannabis*) was detected in 5% of individuals which is lower than the estimation (around 29%), considering that this plant is mainly smoked (i.e. probably less DNA in saliva) and that no accurate survey was available for Papua New Guinea [12]. Tobacco (*Nicotiana*) was detected in 27% of individuals which corresponds to the expected ratio in Papua New Guinea (around 30%) [37]. Overall, our method revealed proportions of the use of these plants close to the surveys.

In order to be conservative, we defined individuals as ‘betel chewers’ if at least 10 fastq reads mapped to both *Areca* and *Piper*, or as ‘non-chewers’ if no read can be mapped to *Areca* or to *Piper*. We identified 50 individuals as ‘betel chewers’ out of 262. This group included 41 men and 9 women, with an average age of 36.7 years, representing all four sampling places (National Capital District, East Sepik Province, Western Province, and Simbu Province). Based on these characteristics and the absence of any read mapping to either *Areca* or *Piper*, we defined a group of 50 ‘non-chewers’ composed by 38 men and 12 women, with an average age of 26.2 years from the four sampling places, in order to have a relative balanced cohort definition for further analyses (Supplementary Table S3). We noted that tobacco is significantly more present in the betel chewers group (*P = *.008; Supplementary Table S3) which can indicate its punctual use in betel nut chewing or common individual behaviour. No difference in cannabis consumption was observed between the two groups (*P* > .05; Supplementary Table S3). Given that our study focussed on the influence of the habit of betel nut chewing on oral microbiome and not *per se* the biochemical effect of *Areca* or *Piper* genera on oral microbiome, we considered the use of tobacco as being punctually part of the habit of betel nut chewing, not defining it as a covariable. The relative low number of tobacco users in our study prevented its analysis separately.

### Microbial diversity in Papua New Guinea

**Figure 1.**
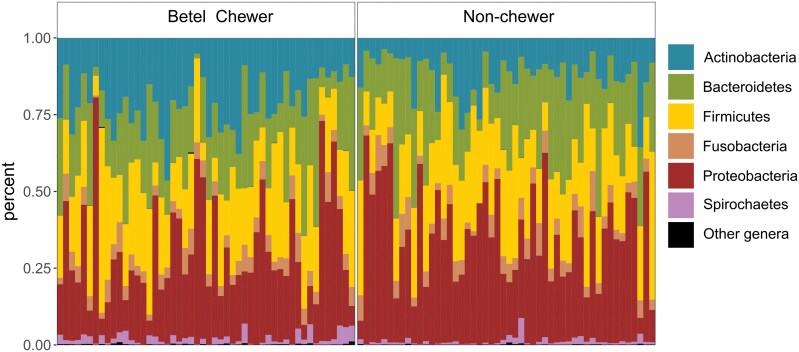
Relative abundances of the six main oral microbial phyla in saliva of betel chewers and non-chewers from Papua New Guinea. Any other phyla are pulled together in ‘other phyla’ category. Each bar corresponds to one individual.

We characterized new oral microbiomes for 100 individuals from Papua New Guinea (Fig. 1 and Supplementary Table S4). Our analysis assigned 62% of reads from shotgun data to a taxon (after removing human reads). Bacterial taxa represented on average 99.8% of the total assigned reads (Supplementary Table S1), while only 0.2% were assigned to viruses, fungi, protozoans, and archaea. The non-bacterial information was not analysed given the limited statistical power. At the individual level, we detected an average of six bacterial phyla, 79 genera and 598 species in the Papua New Guinean oral microbiome, noting that these numbers were likely inflated by the misclassification of rare taxa related to the used method [38].

There is no difference in the number of reads mapping to the human microbiome between the two groups (*P* > .05). In the non-chewers group (i.e. no read mapping on organellar DNA of *Areca* and *Piper*, *n* = 50), the main detected bacterial phyla are: *Proteobacteria* (35%), *Firmicutes* (24%), *Bacteroidetes* (22%), *Actinobacteria* (12%), *Fusobacteria* (5%), and *Spirochaetes* (2%). At the genus level, we found high percentages of *Neisseria* (20%), *Haemophilus* (8%), *Prevotella* (17%), *Veilonella* (6%) *Streptococcus* (14%), *Rothia* (7%), and *Fusobacterium* (5%) (Supplementary Table S4).

In the betel chewers groups (i.e. minimum of 10 reads mapping on organellar DNA of both *Areca* and *Piper*, *n* = 50), the same main phyla were detected. While most of them show similar proportions to those observed in non-chewers, we found a lower relative abundance of *Proteobacteria* (29%) and a higher relative abundance of *Actinobacteria* (21%) (Fig. 1). At the genus level, the main taxa in the betel chewers group are: *Neisseria* (15%), *Prevotella* (15%), *Streptococcus* (11%), *Haemophilus* (9%), *Rothia* (13%), and *Veillonella* (9%) (Supplementary Table S4).

### Influence of betel nut use on the alpha and beta microbial diversities

**Figure 2.**
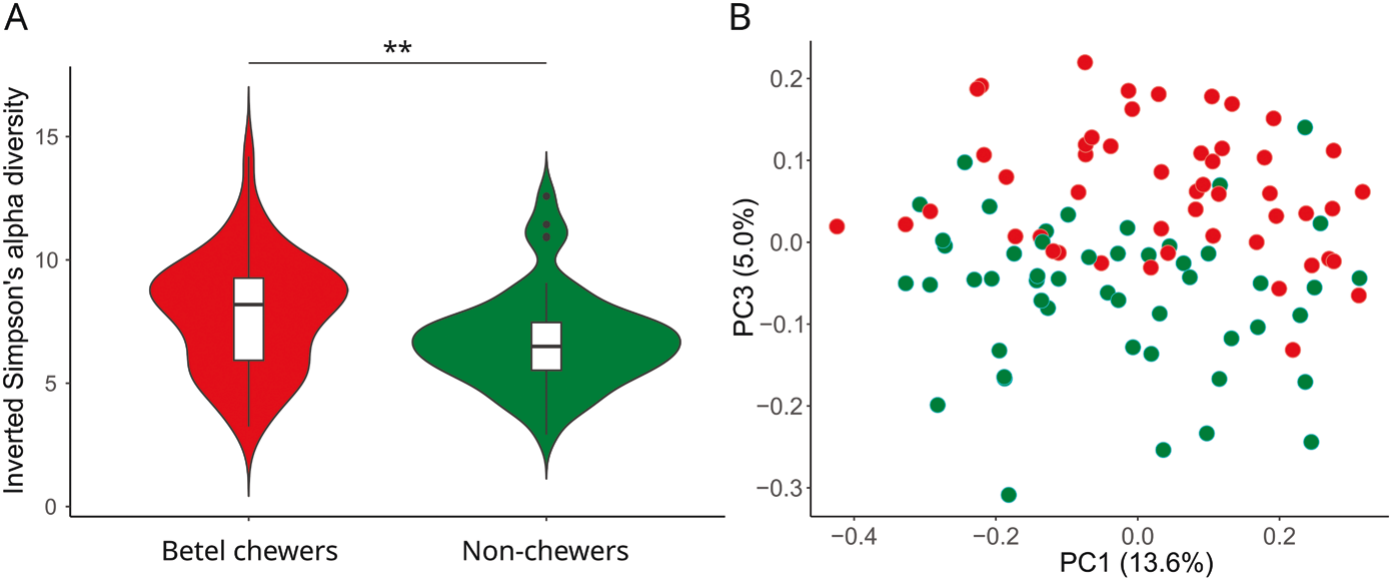
Alpha and beta diversities of the oral microbiome in relation to betel chewing in Papua New Guinea. (A) Violin plot of the Inverse Simpson alpha diversity in the two groups. Stars indicate the level of significance of the *t*-tests (**P > .01). (B) PCoA representing the Bray–Curtis beta diversity of the cohort (principal components 1 and 3).

We calculated the alpha diversity of the oral microbiome for each individual using two indices (Inverse Simpson’s and Shannon’s). For both indices, we found significantly higher alpha diversities in the betel chewers group than in the non-chewers group (Inverse Simpson’s alpha diversity: *t* = 2.52, *P = *.013, *P*_*c*_ = .026; Shannon’s alpha diversity: *t* = 1.97, *P = *.048, *P*_*c*_ > .05; Fig. 2A and Supplementary Fig. S1).

We estimated the beta diversity at the genus level within the cohort and plotted them using different multidimensional scale approaches (NMDS and PCoA; Supplementary Fig. S2). The first two principal components (PC1: 13.6% and PC2: 6.8% of the total variability) tended to separate individual data according to their sampling places (Supplementary Fig. S3), as expected according to our previous study on Papua New Guinean microbial diversity [27]. Other factors such as sequencing batch and depth of reading did not show any pattern according to the first four components (Supplementary Figs. S4 and S5). The third PC (5.0% of the total variability) distinguished beta diversities between betel chewers and non-chewers (Fig. 2B). This apparent clustering is statistically highly significant (*F* = 8.69, *P* < .0001).

### Taxonomic differences related to betel nut chewing

**Figure 3.**
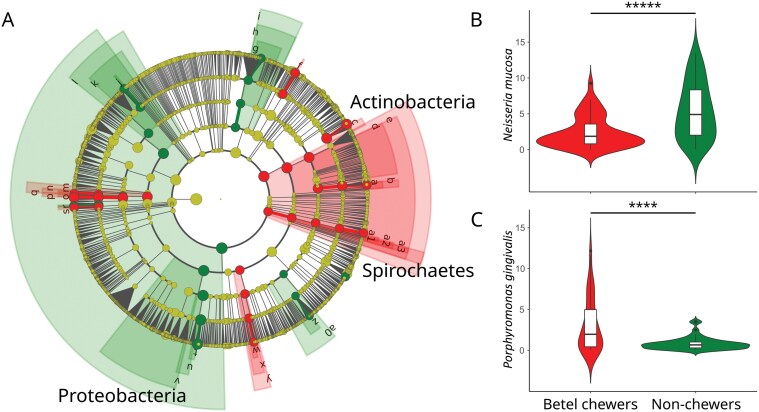
Influence of betel nut chewing in Papua New Guinea on specific bacterial taxa. (A) Cladogram from LEfSe analysis highlighted taxa that were significantly discriminant (LDA score > 2) between the betel chewers group, in red, and the non-chewers group, in green. The hierarchical tree represented the taxonomy, from phyla in the innermost part of the plot to genera in the outermost part. Each circle is a member of the taxonomic level, which size was proportional to its abundance. Shading represents multiple taxa with LDA score > 2 belonging to the same phylotype. a: Actinomycetaceae; b: Actinomycetales; c: Micrococcaceae; d: Micrococcales; e: Actinomycetia; f: Porphyromonadaceae; g: Flavobacteriaceae; h: Flavobacteriales; i: Flavobacteriia; j: Aerococcaceae; k: Bacillales; l: Bacilli; m: Selenomonadaceae; n: Selenomonadales; o: Veillonellaceae; p: Veillonellales; q: Negativicutes; r: Fusobacteriaceae; s: Leptotrichiaceae; t: Neisseriaceae; u: Neisseriales; v: Betaproteobacteria; w: Campylobacteraceae; x: Campylobacterales; y: Epsilonproteobacteria; z: Enterobacteriaceae; a0: Enterobacterales; a1: Spirochaetaceae; a2: Spirochaetales; and a3: Spirochaetia. (B) Violin plot of the relative abundance of *Neisseria mucosa*. (C) Violin plot of the relative abundance of *Porphyromonas gingivalis*. Stars indicate the level of significance of the *t*-tests: ****P = .0001 and *****P = .00001.

We performed a linear discriminant analysis to identify specific microbial genera significantly present in one of the two groups. In relation to betel nut chewing, two main phyla are especially discriminant, with a decrease in proportion of *Proteobacteria* and an increase in proportions of *Actinobacteria* and *Spirochaetes* (|LDA score| > 2; Fig. 3A and Supplementary Fig. S6), confirming our previous observation (Fig. 1). At the genus level, 14 genera are discriminant between the two groups (|LDA score| > 2; Supplementary Fig. S6). In betel chewers, we observed a significantly higher proportion of *Veillonella, Actinomyces, Treponema, Selenomonas, Porphyromonas, Campylobacter, Leptotrichia*, and *Rothia* (Supplementary Table S4). In non-chewers, we observed a significantly higher proportion of *Klebsiella*, *Fusobacterium*, *Neisseria*, *Gemella*, *Aggregatibacter*, and *Capnocytophaga* (Supplementary Table S4).

Within these discriminant bacterial genera, we selected species with a relative abundance of over 0.1% (*n*_taxa_ = 58). Thirty-six taxa showed significant differences of relative abundances between betel chewers and non-chewers (*P*_*c*_ < .05; Supplementary Table S5). We observed a higher relative abundance of discriminant taxa classified as pathogens in betel chewers (12.18%) than in non-chewers (9.57%) (Supplementary Table S5) [32], although some of these species are usually considered commensal in the oral cavity. Six of these discriminant taxa have high relative abundance (abundance > 1%): *Rothia mucilaginosa* (t = 4.39, *P = *.0000031, *P*_*c*_ = .0017; Supplementary Fig. S7), *Fusobacterium pseudoperiodonticum* (*t* = −4.94, *P = *.0000051, *P*_*c*_ = .00029; Supplementary Fig. S7), *Veillonella parvula* (*t* = 5.08, *P = *.000002, *P*_*c*_ = .00018; Supplementary Fig. S7), *Veillonella atypica* (*t* = 3.88, *P = *.0002, *P*_*c*_ = .011; Supplementary Fig. S7), *Neisseria mucosa* (*t* = −4.71, *P = *.00063, *P*_*c*_ = .0004; Fig. 3B), and *Porphyromonas gingivalis* (*t* = 3.59, *P = *.00059, *P*_*c*_ = .034; Fig. 3C). The four latter species are classified as pathogenic. Given the difference observed for *Porphyromonas gingivalis*, we tested differences for two other bacteria often associated with oral diseases [39], and both showed significant increase in betel chewers: *Treponema denticola* (*t* = 3.02, *P = *.0033, *P*_*c*_ > .05; Supplementary Table S5) and *Tannerella forsythia* (*t* = −4.43, *P = *.000039, *P*_*c*_ = .0023; Supplementary Table S5). The relative abundances of *Porphyromonas gingivalis*, *Treponema denticola*, and *Tannerella forsythia,* as well as *Rothia mucilaginosa* and *Fusobacterium pseudoperiodonticum,* were significantly correlated to the third component of the PCoA based on the beta diversity which differentiated betel chewers from non-chewers (Fig. 2B and Supplementary Table S6).

## DISCUSSION

Our study showed that betel nut chewing exerts a strong dysbiosis of the oral microbiome in Papua New Guineans. The oral microbiome of Papua New Guineans is composed of phyla commonly found worldwide [22] in proportions fitting those previously reported in the region [27] (Fig. 1). The oral microbial diversity appeared geographically structured within Papua New Guinea (Supplementary Fig. S3), confirming a previous study [27], with a clear distinction between urban and rural places suggesting a possible impact of urbanization [7]. It is characterized by a relatively high abundance of specific taxa such as *Neisseria* and *Rothia* which was suggested to reflect a regional specificity (Supplementary Tables S4 and S5) [27]. We found that this pattern is significantly altered in betel chewers (Fig. 2). Globally, the oral microbial richness and evenness are increased by betel nut chewing (Fig. 2A), resulting in a relatively homogeneous microbial profile across betel chewers (Fig. 2B). Taxa belonging to the *Proteobacteria* phylum, like *Neisseria mucosa* (lower abundance in betel chewers), and *Actinobacteria* phylum, like *Rothia mucilaginosa* (higher abundance in betel chewers), appeared especially sensitive to betel nut chewing (Fig. 3), which can influence iron homeostasis [40]. The dysbiosis in betel chewers also increased the relative abundance of potential pathogenic bacteria (Supplementary Table S5). It included *Porphyromonas gingivalis* (Fig. 3), *Treponema denticola*, and *Tannerella forsythia* (Supplementary Tables S4 and S5) which form the ‘red complex’ [39]. It defines a co-aggregation and metabolic interdependency among these three bacterial species related to the aetiology of periodontal diseases. A recent paleogenetic analysis in Europe found that *Tannerella forsythia* was drastically impacted by changes in dietary habits over the last millenium [41]. These changes accelerated with industrialization leading to the modification of the virulence repertoire in *Tannerella forsythia* genome, especially in genes coding S-layer proteins that are key factors in the interaction between the host and the pathogen [41]. The habit of betel nut chewing in Papua New Guinea has been present since at least 3000 years and recently increased in the last century [12]. Such a long-term and widespread use of betel nut could have modified the original diversity of the red complex as well as the evolution of its virulence repertoire, ultimately impacting the immune response of chewers. A direct impact of betel nut biochemical components on bacteria was reported [42], but we cannot discard the possibility that our results also depends on other factors linked to the habit. The hunger suppressor effect of betel nut chewing could indirectly lead to oral dysbiosis [43]. Tobacco, which was detected in some betel chewers (Supplementary Table S3), could also impact the oral microbiome [44]. Our results may also have been influenced by the use of genomic datasets not primarily designed for microbiome analysis (e.g. DNA extraction was performed without bacterial cell lysis buffer), favouring the detection of Gram-negative bacterial species, which are commonly found in dysbiosis [45]. While considering these possible cofactors, our results strongly suggested that the habit of betel nut chewing causes a significant dysbiosis of the oral microbiome which could favour the proliferation of important species involved in periodontitis, a major health issue in Papua New Guinea [20].

The association between betel nut chewing and periodontal diseases has been consistently documented in many parts of the world [1, 10, 46, 47]. However, the influence of betel nut chewing on oral microbiome is more complex to interpret globally [11]. While we observed a significantly higher alpha diversity of the oral microbiome in betel chewers, as found in Sri Lanka [23], the opposite was found in India, Pakistan, and Guam [24–26]. Moreover, the bacterial taxa influenced by betel nut chewing are not similar across studies [11]. Even when comparing our results to those from Sri Lanka [23], a higher abundance of bacterial taxa involved in periodontal diseases is found but the species are not fully concordant (*Porphyromonas*, *Treponema*, and *Tannerella* in Papua New Guinea vs. *Actinomyces*, *Tannerella*, and *Prevotella* in Sri Lanka) [23]. Many factors could explain these discrepancies from technical differences (ex: 16S sequencing or shotgun sequencing) to the type of sampling (ex: buccal swab or saliva sampling). Future meta-analyses would benefit from common study designs. In that respect we believe that our approach to genetically identify the plants used in betel nut chewing could be a powerful complementary tool to questionnaires to improve the cohort definition. We could not compare our method to questionnaires on betel nut use, leading to possible pitfalls regarding the detection threshold of plant genetic markers. As it is, our method is likely very conservative, discarding several individual data. The combination of both methods could greatly improve the characterization of betel chewers, and establish the link between the number of betel nut genetic reads in saliva and the amount of used plants. It would also be informative to characterize the correlation between the number of betel nut genetic reads in saliva and the time of the last consumption, and potentially differentiate between occasional and regular consumers. A significant improvement of our method would be to capture and to quantify the genetic signature of all plants in order to obtain a proportion of each species and to explore the quantitative nature of the effect of betel nut chewing on oral microbiome [33]. But outside this global perspective, all of these studies highlight probable regional specificities of betel nut chewing and their impact on the oral microbiome. They could be the reflect of specific human oral microbial diversities [7], different sociocultural behaviour related to betel nut chewing [1], and even different biomolecular compositions of the *Areca* nut itself [48]. Specificities within a region should also be considered. For example, in Papua New Guinea, inter-population oral microbial differences were suggested [27], different uses of betel nut were documented across cultures [12] and two types of *Areca* nuts and *Piper* fruits are used, with distinct origins [12, 14] and potentially biochemical properties [49]. This complexity could lead to diverse influences of betel nut chewing on human oral microbiome within Papua New Guinea. The biocultural context thus needs to be considered to characterize the interaction between human biology and plants.

Our study strongly suggested that betel nut chewing in Papua New Guinea has a detrimental impact on human oral microbiome homeostasis, which could ultimately lead to periodontal diseases. Our study has some limitations such as the absence of questionnaires on betel nut chewing habit, the moderate number of participants, and the use of a publicly available dataset not optimized for microbiome analysis. However, it paved the way towards a better understanding of the impact of betel nut on Papua New Guinean biology, and it presented a novel and complementary approach to characterize betel nut chewing that can be extended to other regions of the world. Given the difficulty to control the population’s habits of betel nut chewing and the current increase of oral diseases in Papua New Guinea [50], our study advocates to limit its use and supports more education and awareness programs about the risks of betel nut consumption.

## Supplementary Material

eoae030_suppl_Supplementary_Materials

eoae030_suppl_Supplementary_Tables

## Data Availability

All data used in this study have been deposited in the European Genome-phenome Archive under accession codes EGAD00001010142 (https://ega-archive.org/datasets/EGAD00001010142), EGAD00001010143 (https://ega-archive.org/datasets/EGAD00001010143), EGAD50000000050 (https://ega-archive.org/datasets/EGAD50000000050) and EGAD00001007783 (https://ega-archive.org/datasets/EGAD00001007783). All codes used in this study are available at: https://github.com/nbrucato/Microbiome/tree/main.
