# Supplementary material for: Influence of betel nut chewing on oral microbiome in Papua New Guinea
Source: Evol Med Public Health. 2024 Nov 9;13(1):36–44. doi: 10.1093/emph/eoae030 (PMC11928724; doi:10.1093/emph/eoae030)
Supplement: eoae030_suppl_Supplementary_Materials [file eoae030_suppl_supplementary_materials.docx]

**SUPPLEMENTARY FIGURES**


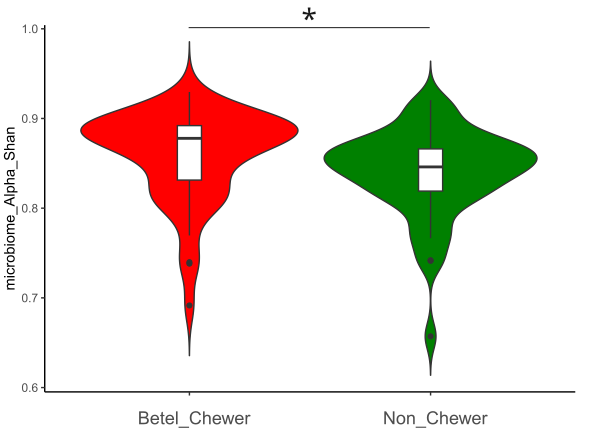


**Figure S1.** Violin plot of the Shannon alpha diversity index in Betel chewers and Non-chewers. *: *p* < 0.05.


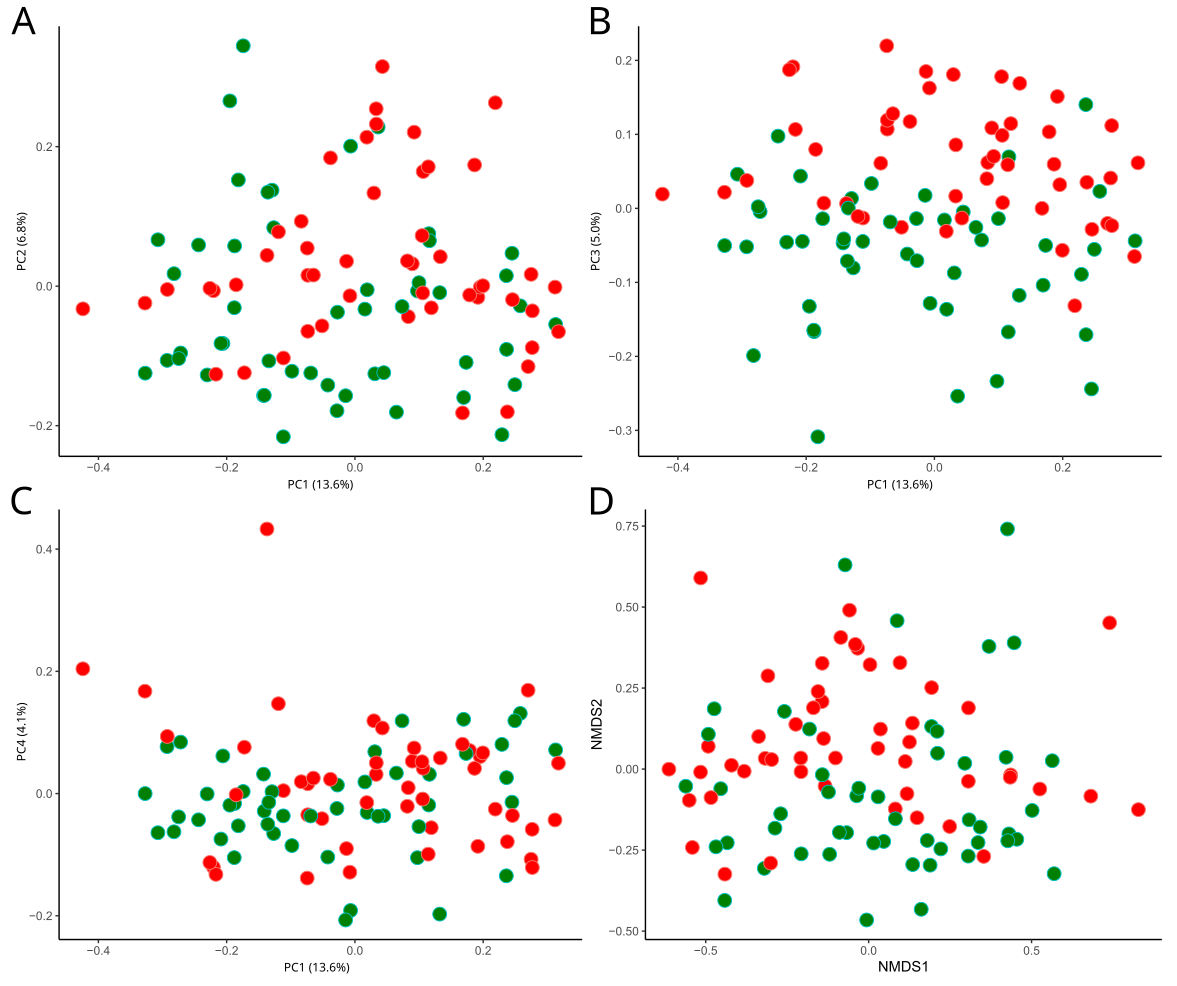


**Figure S2.** Oral microbiome beta ‘Bray-Curtis’ diversity in Papua New Guinea represented by PCoA plots: (A) PC1-PC2, (B) PC1-PC3, (C) PC1-PC4; and by (D) NMDS plot (Dimensions 1 and 2). Red dots represent betel chewers and green dots represent non-chewers.

**
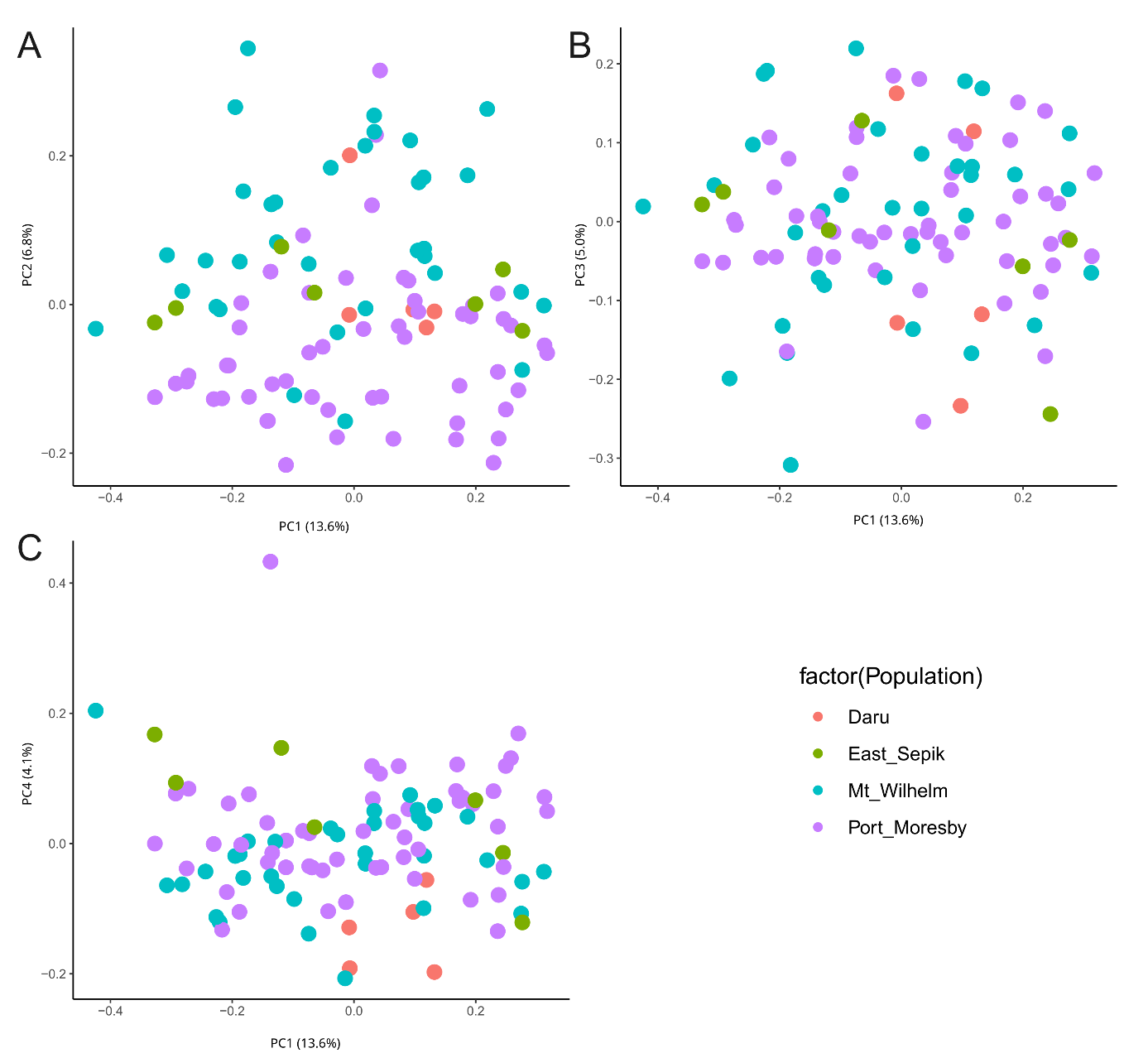
**

**Figure S3.** Oral microbiome beta ‘Bray-Curtis’ diversity in Papua New Guinea represented by PCoA plots: (A) PC1-PC2, (B) PC1-PC3, (C) PC1-PC4. Dots are coloured according to the sampling places.

**
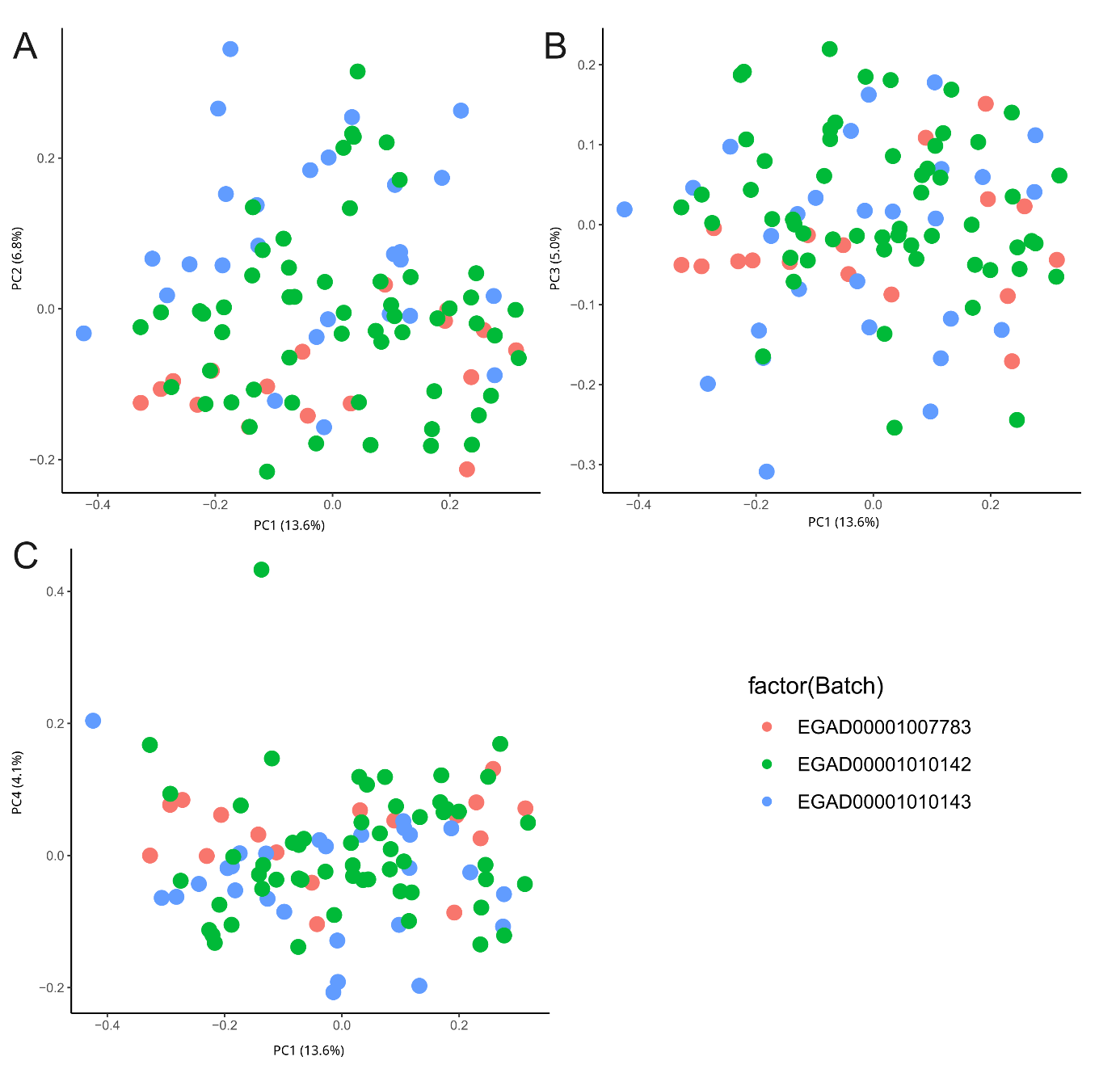
**

**Figure S4.** Oral microbiome beta ‘Bray-Curtis’ diversity in Papua New Guinea represented by PCoA plots: (A) PC1-PC2, (B) PC1-PC3, (C) PC1-PC4. Dots are coloured according to the sequencing batches. The batch EGAD50000000050 was not represented in the selected set of individuals analysed in the study.

**
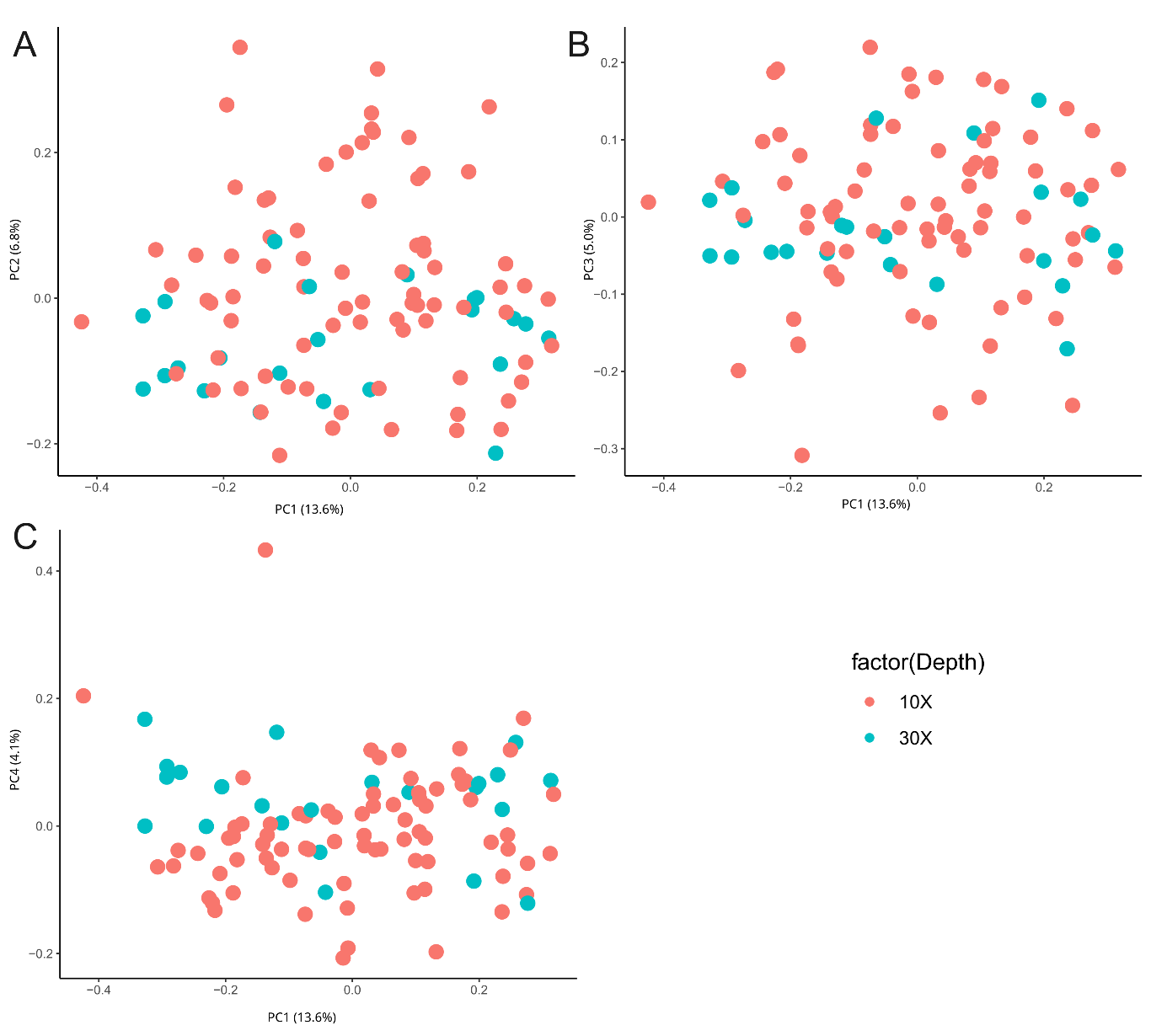
**

**Figure S5.** Oral microbiome beta ‘Bray-Curtis’ diversity in Papua New Guinea represented by PCoA plots: (A) PC1-PC2, (B) PC1-PC3, (C) PC1-PC4. Dots are coloured according to the depth of reading of the sequencing data.


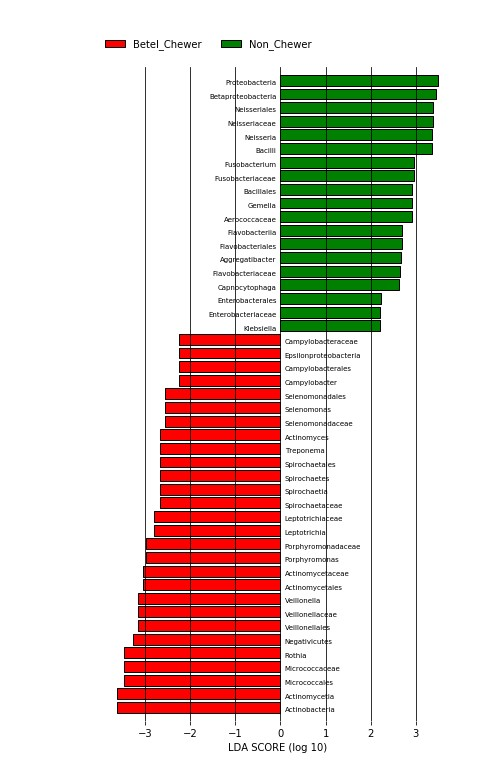


**Figure S6.** Linear Discriminant Analysis of the oral microbiome between Betel chewers and Non-chewers. Only |LDA scores| > 2 are shown.

**
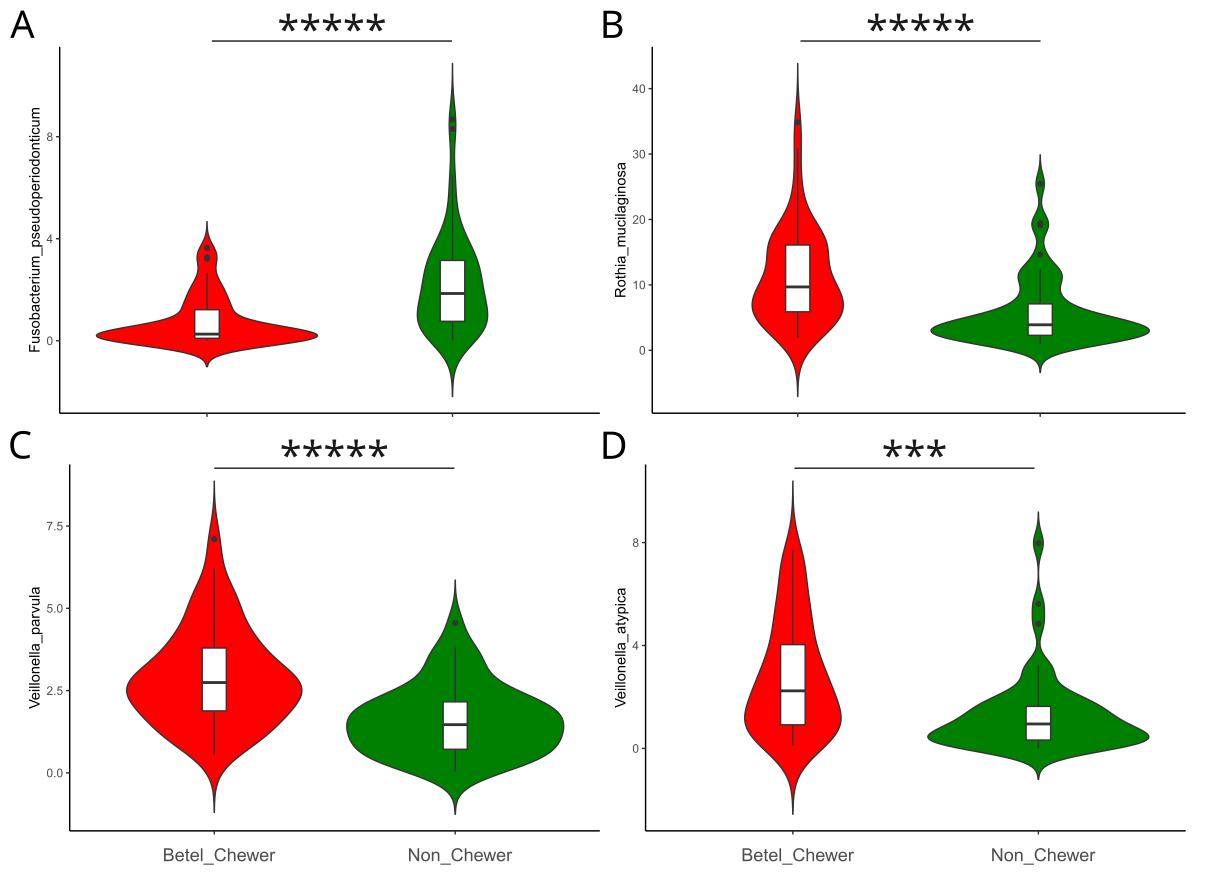
**

**Figure S7.** Relative abundance of (A) *Fusobacterium pseudoperiodonticum,* (B) *Rothia mucilaginosa*, (C) *Veillonella atypica*, and (D) *Veillonella parvula* between betel chewers (in red) and non-chewers (in green). *****: *p* < 0.00005; ***: *p* < 0.005.
